# Schaurteite, Ca_3_Ge(SO_4_)_2_(OH)_6_·3H_2_O

**DOI:** 10.1107/S1600536812050945

**Published:** 2013-01-12

**Authors:** Marcus J. Origlieri, Robert T. Downs

**Affiliations:** a Department of Geosciences, University of Arizona, Tucson, AZ 85721, USA

## Abstract

This report presents the first crystal structure determination of the mineral schaurteite, ideally Ca_3_Ge(SO_4_)_2_(OH)_6_·3H_2_O, tricalcium germanium bis­(sulfate) hexa­hydroxide trihydrate. This single-crystal X-ray diffraction study investigated a natural sample from the type locality at Tsumeb, Namibia. Schaurteite is a member of the fleischerite group of minerals, which also includes fleischerite, despujolsite, and mallestigite. The structure of schaurteite consists of slabs of Ca(O,OH,H_2_O)_8_ polyhedra (site symmetry *mm*2) inter­leaved with a mixed layer of Ge(OH)_6_ octa­hedra (-3*m*.) and SO_4_ tetra­hedra (3*m*.). There are two H atoms in the asymmetric unit, both located by full-matrix refinement, and both forming O—H⋯O hydrogen bonds.

## Related literature

For the original description of schaurteite, see: Strunz & Tennyson (1967 ▶). For descriptions of related minerals: fleischerite (Frondel & Strunz, 1960 ▶); despujolsite (Gaudefroy *et al.*, 1968 ▶); mallestigite (Sima *et al.*, 1996 ▶). For structural refinements of related minerals: despujolsite (Barkley *et al.*, 2011 ▶); fleischerite (Otto, 1975 ▶). For analysis of anisotropic displacement parameters, see: Downs (2000 ▶).

## Experimental

### 

#### Crystal data

Ca_3_Ge(SO_4_)_2_(OH)_6_(H_2_O)_3_
*M* *_r_* = 541.05
Hexagonal, 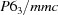

*a* = 8.5253 (4) Å
*c* = 10.8039 (6) Å
*V* = 680.03 (6) Å^3^
*Z* = 2
Mo *K*α radiation
μ = 3.79 mm^−1^
*T* = 296 K
0.05 × 0.03 × 0.03 mm

#### Data collection

Bruker APEXII CCD diffractometer
Absorption correction: multi-scan (*SADABS*; Sheldrick, 2005 ▶) *T*_min_ = 0.656, *T*_max_ = 0.747
17243 measured reflections
536 independent reflections
456 reflections with *I* > 2σ(*I*)
*R*_int_ = 0.061

#### Refinement

*R*[*F* ^2^ > 2σ(*F* ^2^)] = 0.022
*wR*(*F* ^2^) = 0.045
*S* = 1.15
536 reflections
35 parameters
All H-atom parameters refined
Δρ_max_ = 0.40 e Å^−3^
Δρ_min_ = −0.42 e Å^−3^

### 

Data collection: *APEX2* (Bruker, 2004 ▶); cell refinement: *SAINT* (Bruker, 2005 ▶); data reduction: *SAINT*; program(s) used to solve structure: *SHELXS97* (Sheldrick, 2008 ▶); program(s) used to refine structure: *SHELXL97* (Sheldrick, 2008 ▶); molecular graphics: *XtalDraw* (Downs & Hall-Wallace, 2003 ▶); software used to prepare material for publication: *SHELXL97*.

## Supplementary Material

Crystal structure: contains datablock(s) I, global. DOI: 10.1107/S1600536812050945/br2217sup1.cif

Structure factors: contains datablock(s) I. DOI: 10.1107/S1600536812050945/br2217Isup2.hkl

Supplementary material file. DOI: 10.1107/S1600536812050945/br2217Isup3.cml

Additional supplementary materials:  crystallographic information; 3D view; checkCIF report

## Figures and Tables

**Table 1:** Hydrogen-bond geometry (Å, °)

*D*—H⋯*A*	*D*—H	H⋯*A*	*D*⋯*A*	*D*—H⋯*A*
O3—H1⋯O2^i^	0.73 (3)	2.12 (3)	2.823 (2)	164 (3)
O4—H2⋯O1^ii^	0.79 (3)	2.04 (3)	2.789 (2)	158 (3)

Symmetry codes: (i) 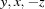; (ii) 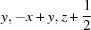.

**Fig. 1.**
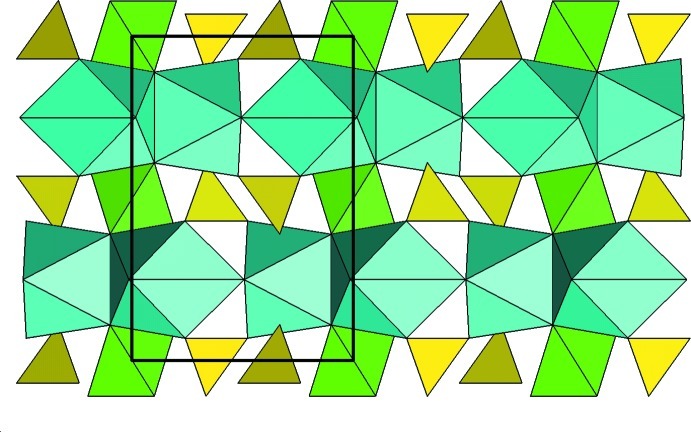
Polyhedral view of the schaurteite structure down [100], with slabs of light blue Ca(OH)4O2(H2O)2 polyhedra, interleaved with a mixed layer of green Ge(OH)6 octahedra and yellow SO4 tetrahedra. Unit cell outline shown in black.

**Fig. 2.**
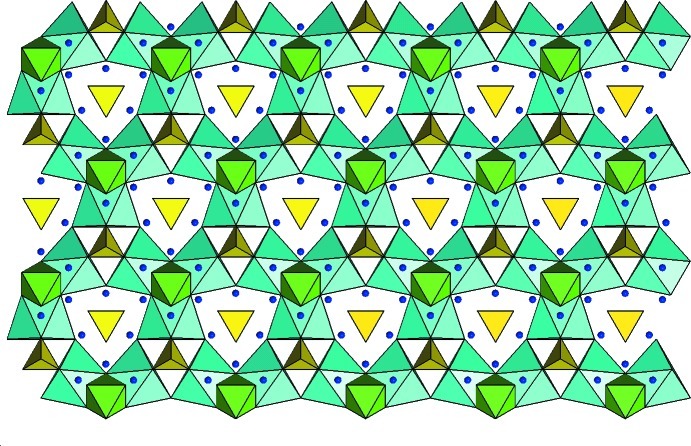
Polyhedral view of the schaurteite structure down [001], showing a single layer of light blue Ca(OH)4O2(H2O)2 polyhedra, a single mixed layer of green Ge(OH)6 octahedra and yellow SO4 tetrahedra, and dark blue H atoms in both layers.

**Fig. 3.**
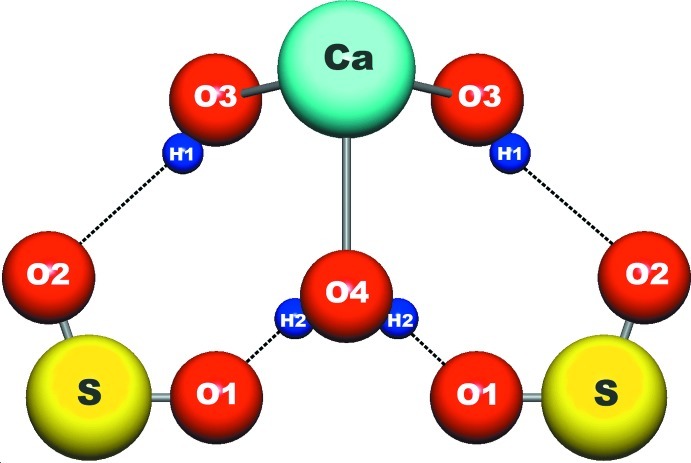
Hydrogen bonding in schaurteite, showing O4—H2···O1 at 2.04 Å and O3—H1···O2 at 2.12 Å.

**Fig. 4.**
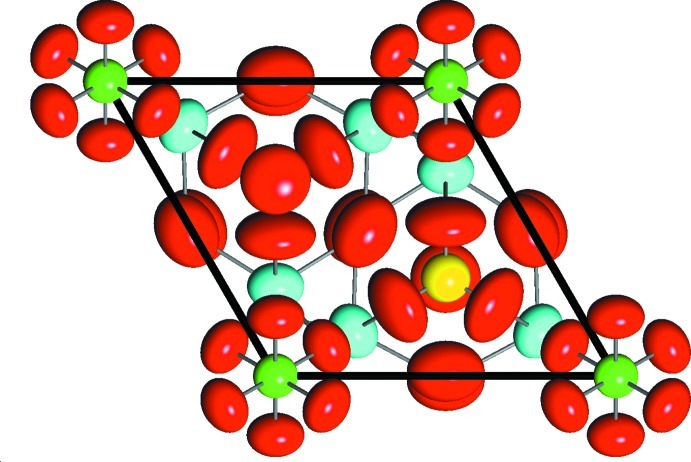
Ellipsoidal view of the schaurteite structure down [001], showing red O atoms, yellow S atoms, green Ge atoms, and light blue Ca atoms. Unit cell outline shown in black. The large circular ellipsoid at [2/3,1/3,z] represents the O1 atom (3 m. symmetry), which forms the apex of a rigid SO4 tetrahedron with significant translation (0.73 Å) and libration (7.97°).

## supplementary crystallographic information

### Comment

Schaurteite, Ca_3_Ge(SO_4_)_2_(OH)_6_.3H_2_O, is a rare germanium mineral found in oxidized germanite ores at the Tsumeb Corporation mine, Tsumeb, Namibia (Strunz & Tennyson, 1967). Schaurteite belongs to the fleischerite group of isotypic minerals, along with mallestigite, Pb_3_Sb(SO_4_)(AsO_4_)(OH)_6_.3H_2_O (Sima *et al.* 1996), despujolsite, Ca_3_Mn^4+^(SO_4_)_2_(OH)_6_.3H_2_O (Gaudefroy *et al.* 1968), and fleischerite, Pb_3_Ge(SO_4_)_2_(OH)_6_.3H_2_O (Frondel & Strunz, 1960). Of these four minerals, only despujolsite (Barkley *et al.* 2011) and synthetic fleischerite (Otto, 1975) have reported structures. This study represents the first structural report for schaurteite.

The crystal structure of schaurteite consists of slabs of Ca(OH)_4_O_2_(H_2_O)_2_ polyhedra (*mm.* symmetry), interconnected by mixed layers of Ge(OH)_6_ octahedra (3*m*. symmetry) and SO_4_ tetrahedra (3*m*. symmetry) (Figures 1,2). The mean Ca—O, Ge—O, and S—O bond lengths are 2.487 Å, 1.895 Å, and 1.468 Å, respectively. There are two separate hydrogen atoms, H1 bonded to the O3 atom coordinating Ge, and H2 bonded to the O4 atom (*mm*. symmetry) which generates an H_2_O molecule. Both H atoms form hydrogen bonds, O3—H1···O2 and O4—H2···O1 (Figure 3), mimicking those seen in despujolsite (Barkley *et al.* 2011).

In the original description of schaurteite, Strunz and Tennyson (1967) noted systematic absences in X-ray photographs consistent with three different space groups: *P*6_3_*/mmc*, *P*6_3_*mc*, and *P*62*c*. Supposing an isostructural relationship with despujolsite (Barkley *et al.* 2011), this study initially refined schaurteite in space group *P*62*c* with a favorable *R*_obs_ of 0.0251 for 45 parameters. Despite the relatively low *R* factor, the positional parameters for H2 did not converge, and a twin model showed a racemic component of 47%, indicating centrosymmetry in the structure. In space group *P*6_3_*/mmc*, the structural refinement converged rapidly for all atomic coordinates including those for H, and *R*_obs_ dropped to .0219 for 35 parameters. Consequently, this study proposes *P*6_3_*/mmc* symmetry for schaurteite, in contrast to *P*62*c* symmetry reported for both fleischerite (Otto, 1975) and despujolsite (Barkley *et al.* 2011). Curiously, schaurteite, Ca_3_Ge(SO_4_)_2_(OH)_6_.3H_2_O, and fleischerite, Pb_3_Ge(SO_4_)_2_(OH)_6_.3H_2_O, are both single locality mineral species occurring at the same deposit, notably lacking significant solid solution between Pb and Ca in their reported analyses (Strunz and Tennyson, 1967; this study; Frondel and Strunz, 1960). If unbonded lone pair electrons belonging to Pb in fleischerite disrupt the centrosymmetry seen in schaurteite, this would explain an ostensibly limited solid solution between fleischerite and schaurteite.

Previous studies of fleischerite group minerals noted relatively large displacement parameters for O atoms in the SO_4_ tetrahedron. For this reason, Otto (1975) proposed a split site for the O1 atom in the structure of synthetic fleischerite. When Barkley *et al.* (2011) reported the structure of the isotypic Mn^4+^ analog despujolsite, they also noted large displacement parameters for O1. However, Barkley *et al.* (2011) proposed a single O1 site for despujolsite after an analysis of the displacement parameters demonstrated that the SO_4_ group behaves as a rigid body with significant translation (0.72 Å) and libration (7.95°). Using translation-libration-screw motion (TLS) modelling software (Downs, 2000), the SO_4_ group in schaurteite similarly shows rigid body behavior with 0.73 Å of translation and 7.97° of libration. Consequently, this study proposes a single O1 atom model for schaurteite (Figure 4).

### Experimental

The schaurteite specimen used in this study came from the Tsumeb Corporation mine, Tsumeb, Otavi Mountains, Namibia and remains on deposition (sample R120104) with the RRUFF project (http://www.rruff.info). Schaurteite forms colorless fibrous crystals in a matrix of quartz, calcite, and germanite.

Chemical analyses were performed on a Cameca SX-100 electron microprobe at the Lunar and Planetary Laboratory, University of Arizona. The electron microprobe sample and the single-crystal fragment came from the same parent sample. Like despujolsite (Barkley *et al.* 2011), schaurteite was fugitive under the electron beam, leading to the choice of the following operating conditions: 20 kV exciting voltage, 6 nA operating current, and a spot size of 5 microns. The average of 13 analyses gave GeO_2_ 17.97%, CaO 30.41%, and SO_3_ 29.22%. Normalizing to 17 O atoms (which includes 6 moles H_2_O per formula unit), the combined empirical and structural chemical formula becomes Ca_3.01_Ge_0.95_(S_2.03_O_8_)(OH)_6_.3H_2_O. Further details of the electron microprobe analysis and formula calculations are available on the RRUFF web site (http://www.rruff.info/R120104).

### Refinement

To model possible variable occupancy of cations, site occupancies were allowed to vary without restraints. The results justified a model with full occupancies of Ca, Ge, and S, corroborated by electron microprobe data showing only these elements (with *Z* > 8). Two hydrogen atom positions were refined without restraints, both with reasonable isotropic displacement parameters.

### Crystal data

**Table:**

Ca_3_Ge(SO_4_)_2_(OH)_6_(H_2_O)_3_	*D*_x_ = 2.642 Mg m^−^^3^
*M**_r_* = 541.05	Mo *K*α radiation, λ = 0.71073 Å
Hexagonal, *P*6_3_/*m**m**c*	Cell parameters from 2603 reflections
Hall symbol: -P 6c 2c	θ = 2.8–28.0°
*a* = 8.5253 (4) Å	µ = 3.79 mm^−^^1^
*c* = 10.8039 (6) Å	*T* = 296 K
*V* = 680.03 (6) Å^3^	Hexagonal prism section, colourless
*Z* = 2	0.05 × 0.03 × 0.03 mm
*F*(000) = 544	

### Data collection

**Table:**

Bruker APEXII CCD diffractometer	536 independent reflections
Radiation source: fine-focus sealed tube	456 reflections with *I* > 2σ(*I*)
Graphite monochromator	*R*_int_ = 0.061
φ and ω scans	θ_max_ = 33.1°, θ_min_ = 2.8°
Absorption correction: multi-scan (*SADABS*; Sheldrick, 2005)	*h* = −10→13
*T*_min_ = 0.656, *T*_max_ = 0.747	*k* = −13→13
17243 measured reflections	*l* = −16→16

### Refinement

**Table:**

Refinement on *F*^2^	Primary atom site location: structure-invariant direct methods
Least-squares matrix: full	Secondary atom site location: difference Fourier map
*R*[*F*^2^ > 2σ(*F*^2^)] = 0.022	Hydrogen site location: difference Fourier map
*wR*(*F*^2^) = 0.045	All H-atom parameters refined
*S* = 1.15	*w* = 1/[σ^2^(*F*_o_^2^) + (0.0131*P*)^2^ + 0.5886*P*] where *P* = (*F*_o_^2^ + 2*F*_c_^2^)/3
536 reflections	(Δ/σ)_max_ < 0.001
35 parameters	Δρ_max_ = 0.40 e Å^−^^3^
0 restraints	Δρ_min_ = −0.42 e Å^−^^3^

### Special details

**Table:**

Geometry. All esds (except the esd in the dihedral angle between two l.s. planes) are estimated using the full covariance matrix. The cell esds are taken into account individually in the estimation of esds in distances, angles and torsion angles; correlations between esds in cell parameters are only used when they are defined by crystal symmetry. An approximate (isotropic) treatment of cell esds is used for estimating esds involving l.s. planes.
Refinement. Refinement of *F*^2^ against ALL reflections. The weighted *R*-factor *wR* and goodness of fit *S* are based on *F*^2^, conventional *R*-factors *R* are based on *F*, with *F* set to zero for negative *F*^2^. The threshold expression of *F*^2^ > σ(*F*^2^) is used only for calculating *R*-factors(gt) *etc*. and is not relevant to the choice of reflections for refinement. *R*-factors based on *F*^2^ are statistically about twice as large as those based on *F*, and *R*- factors based on ALL data will be even larger.

### Fractional atomic coordinates and isotropic or equivalent isotropic displacement parameters (Å^2^)

**Table:**

	*x*	*y*	*z*	*U*_iso_*/*U*_eq_	
Ge	0.0000	0.0000	0.0000	0.00762 (11)	
Ca	0.30387 (8)	0.15193 (4)	0.2500	0.01107 (12)	
S	0.3333	0.6667	0.52599 (7)	0.00963 (16)	
O1	0.3333	0.6667	0.1106 (2)	0.0167 (5)	
O2	0.4786 (2)	0.23930 (11)	0.06925 (14)	0.0190 (3)	
O3	0.10006 (10)	0.2001 (2)	0.10982 (13)	0.0106 (3)	
O4	0.50772 (16)	0.49228 (16)	0.7500	0.0190 (5)	
H1	0.142 (2)	0.284 (4)	0.075 (2)	0.028 (8)*	
H2	0.543 (2)	0.457 (2)	0.695 (3)	0.051 (11)*	

### Atomic displacement parameters (Å^2^)

**Table:**

	*U*^11^	*U*^22^	*U*^33^	*U*^12^	*U*^13^	*U*^23^
Ge	0.00878 (15)	0.00878 (15)	0.0053 (2)	0.00439 (7)	0.000	0.000
Ca	0.0098 (3)	0.0131 (2)	0.0091 (2)	0.00491 (13)	0.000	0.000
S	0.0108 (2)	0.0108 (2)	0.0073 (3)	0.00538 (11)	0.000	0.000
O1	0.0217 (9)	0.0217 (9)	0.0067 (11)	0.0109 (4)	0.000	0.000
O2	0.0114 (7)	0.0243 (7)	0.0169 (7)	0.0057 (4)	0.0039 (6)	0.0020 (3)
O3	0.0131 (5)	0.0086 (7)	0.0087 (6)	0.0043 (3)	−0.0004 (3)	−0.0008 (5)
O4	0.0239 (9)	0.0239 (9)	0.0143 (10)	0.0158 (10)	0.000	0.000

### Geometric parameters (Å, º)

**Table:**

Ge—O3	1.8949 (14)	Ca—O3^iii^	2.4890 (9)
Ge—O3^i^	1.8949 (14)	Ca—O3^vii^	2.4890 (9)
Ge—O3^ii^	1.8949 (14)	Ca—O4^viii^	2.6284 (12)
Ge—O3^iii^	1.8949 (14)	Ca—O4^ix^	2.6284 (12)
Ge—O3^iv^	1.8949 (14)	S—O2^x^	1.4651 (16)
Ge—O3^v^	1.8949 (14)	S—O2^xi^	1.4651 (16)
Ca—O2	2.3404 (15)	S—O2^xii^	1.4651 (16)
Ca—O2^vi^	2.3404 (15)	S—O1^vi^	1.475 (3)
Ca—O3	2.4890 (9)	O3—H1	0.73 (3)
Ca—O3^vi^	2.4890 (9)	O4—H2	0.79 (3)
			
O3—Ge—O3^i^	180.0	O2—Ca—O3^vii^	147.24 (3)
O3—Ge—O3^ii^	95.05 (6)	O2^vi^—Ca—O3^vii^	79.95 (4)
O3^i^—Ge—O3^ii^	84.95 (6)	O3—Ca—O3^vii^	105.61 (6)
O3—Ge—O3^iii^	84.95 (6)	O3^vi^—Ca—O3^vii^	61.87 (7)
O3^i^—Ge—O3^iii^	95.05 (6)	O3^iii^—Ca—O3^vii^	74.96 (5)
O3^ii^—Ge—O3^iii^	180.00 (10)	O2—Ca—O4^viii^	73.04 (3)
O3—Ge—O3^iv^	95.05 (6)	O2^vi^—Ca—O4^viii^	73.04 (3)
O3^i^—Ge—O3^iv^	84.95 (6)	O3—Ca—O4^viii^	83.33 (5)
O3^ii^—Ge—O3^iv^	84.95 (6)	O3^vi^—Ca—O4^viii^	83.33 (5)
O3^iii^—Ge—O3^iv^	95.05 (6)	O3^iii^—Ca—O4^viii^	139.12 (3)
O3—Ge—O3^v^	84.95 (6)	O3^vii^—Ca—O4^viii^	139.12 (3)
O3^i^—Ge—O3^v^	95.05 (6)	O2—Ca—O4^ix^	73.04 (3)
O3^ii^—Ge—O3^v^	95.05 (6)	O2^vi^—Ca—O4^ix^	73.04 (3)
O3^iii^—Ge—O3^v^	84.95 (6)	O3—Ca—O4^ix^	139.12 (3)
O3^iv^—Ge—O3^v^	180.00 (14)	O3^vi^—Ca—O4^ix^	139.12 (3)
O2—Ca—O2^vi^	113.10 (8)	O3^iii^—Ca—O4^ix^	83.33 (5)
O2—Ca—O3	79.95 (4)	O3^vii^—Ca—O4^ix^	83.33 (5)
O2^vi^—Ca—O3	147.24 (3)	O4^viii^—Ca—O4^ix^	116.09 (10)
O2—Ca—O3^vi^	147.24 (3)	O2—Ca—Ge	73.16 (4)
O2^vi^—Ca—O3^vi^	79.95 (4)	O2^x^—S—O2^xi^	110.32 (7)
O3—Ca—O3^vi^	74.96 (5)	O2^x^—S—O2^xii^	110.32 (7)
O2—Ca—O3^iii^	79.95 (4)	O2^xi^—S—O2^xii^	110.32 (7)
O2^vi^—Ca—O3^iii^	147.24 (3)	O2^x^—S—O1^vi^	108.61 (7)
O3—Ca—O3^iii^	61.87 (7)	O2^xi^—S—O1^vi^	108.60 (7)
O3^vi^—Ca—O3^iii^	105.61 (6)	O2^xii^—S—O1^vi^	108.60 (7)

Symmetry codes: (i) −*x*, −*y*, −*z*; (ii) *x*−*y*, *x*, −*z*; (iii) −*x*+*y*, −*x*, *z*; (iv) *y*, −*x*+*y*, −*z*; (v) −*y*, *x*−*y*, *z*; (vi) *x*, *y*, −*z*+1/2; (vii) −*x*+*y*, −*x*, −*z*+1/2; (viii) −*x*+1, −*y*+1, −*z*+1; (ix) *y*, −*x*+*y*, −*z*+1; (x) *y*, −*x*+*y*+1, *z*+1/2; (xi) −*x*+1, −*y*+1, *z*+1/2; (xii) *x*−*y*, *x*, *z*+1/2.

### Hydrogen-bond geometry (Å, º)

**Table:**

*D*—H···*A*	*D*—H	H···*A*	*D*···*A*	*D*—H···*A*
O3—H1···O2^xiii^	0.73 (3)	2.12 (3)	2.823 (2)	164 (3)
O4—H2···O1^xiv^	0.79 (3)	2.04 (3)	2.789 (2)	158 (3)

Symmetry codes: (xiii) *y*, *x*, −*z*; (xiv) *y*, −*x*+*y*, *z*+1/2.
